# Adverse Effects of Anti-PD-1/PD-L1 Therapy in Non-small Cell Lung Cancer

**DOI:** 10.3389/fonc.2020.554313

**Published:** 2020-09-17

**Authors:** Chaoyue Su, Hui Wang, Yunru Liu, Qiaoru Guo, Lingling Zhang, Jiajun Li, Wenmin Zhou, Yanyan Yan, Xinke Zhou, Jianye Zhang

**Affiliations:** ^1^The Fifth Affiliated Hospital, Key Laboratory of Molecular Target and Clinical Pharmacology and the State Key Laboratory of Respiratory Disease, School of Pharmaceutical Sciences, Guangzhou Medical University, Guangzhou, China; ^2^School of Public Health, Hainan Medical University, Haikou, China; ^3^Guangzhou Institute of Pediatrics/Guangzhou Women and Children's Medical Center, Guangzhou Medical University, Guangzhou, China; ^4^Institute of Immunology and School of Medicine, Shanxi Datong University, Datong, China

**Keywords:** adverse effects, PD-1/PD-L1, immunotherapy, immune checkpoint inhibitor, non-small cell lung cancer

## Abstract

Currently, immunotherapy has shown great efficacy in clinical trials, and monoclonal antibodies directed against immune checkpoint PD-1/PD-L1 have shown encouraging results in first-line or second-line treatment of non-small cell lung cancer patients. Meanwhile, anti-PD-1/PD-L1 immune checkpoint drugs combined with other treatments, such as chemotherapy, targeted therapy as well as anti-CTLA-4 checkpoint therapy, are considered an attractive treatment with higher efficacy. However, toxicity associated with PD-1/PD-L1 blockade is worth attention. Understanding the adverse effects caused by anti-PD-1/PD-L1 immunosuppressive agents is vital to guide the clinical rational use of drug. In this review, we summarized the adverse effects that occurred during the clinical use of anti-PD-1/PD-L1 inhibitors in the treatment of non-small cell lung cancer and discussed how to effectively manage and respond to these adverse reactions.

## Introduction

Currently, cancer is still a key threat to human health (1). Among them, lung cancer is the leading cause of cancer-related deaths worldwide, and about 80% of lung cancer is non-small cell lung cancer (NSCLC), with poor prognosis (2, 3). Encouragingly, the blockade of immune checkpoints against PD-1/PD-L1 has dramatically changed the treatment prospects for patients with NSCLC (4–6). The traditional treatments of cancer are mainly target at the cancer cells themselves, while the main goal of tumor immunotherapy is to enhance or restore the monitoring and killing effect of the body's immune system on tumors (7–9). There are many immune checkpoint molecules in the body, which are involved in maintaining the body's immune balance and its own immune tolerance (10). Among them, PD-1 and cytotoxic T lymphocyte-associated protein 4 (CTLA-4) are classic co-inhibitory molecules that suppress the immune response (11–13). Tumor cells overexpress the immunosuppressive surface ligand PD-L1, which interacts with T cell molecules, leading to T cell failure (14, 15). Knowledge based on the immune escape mechanism of cancer cells has led to the development of immunological checkpoint inhibitors (16, 17).

In recent years, immune checkpoint inhibitors (ICIs) have been widely used in tumor immunotherapy (18, 19). ICIs based on the PD-1/PD-L1 axis have been proved to exhibit promising therapeutic effects in a variety of advanced cancers (20–22). For example, the anti-PD-1 ICIs nivolumab and pembrolizumab have shown exciting results in the treatment of metastatic melanoma and NSCLC (23, 24). Moreover, anti-PD-L1 antibody durvalumab, atezolizumab as well as avelumab have also shown anti-tumor activity in a number of tumor types. However, it is worth noting that as the immune system is reactivated, the body's immune tolerance imbalance occurs (10). Immunotherapy leads to the emergence of novel toxic features, known as immune-related adverse events (irAEs), by reactivating the immune system (14). Although severe irAEs are rare, they can be life-threatening without intervention and proper management (25, 26). In addition, it has also been reported that the combined use of PD-1/PD-L1 ICIs with chemotherapeutics or other targeted therapies leads to the emergence of new toxic reactions (14). Therefore, raising our awareness of these adverse events (AEs) is critical to optimize the clinical efficacy and safety of these new immunotherapeutic.

In this review, we summarized the adverse reactions of the five FDA-approved targeted PD-1/PD-L1 immune checkpoint drugs currently used in the clinic when used alone or in combination with other treatments in NSCLC patients. We aim to raise awareness of the clinical manifestations, diagnosis, and management of these toxic reactions through our summary.

## Mechanism Overview of PD-1/PD-L1 Blockade

**Figure 1:**
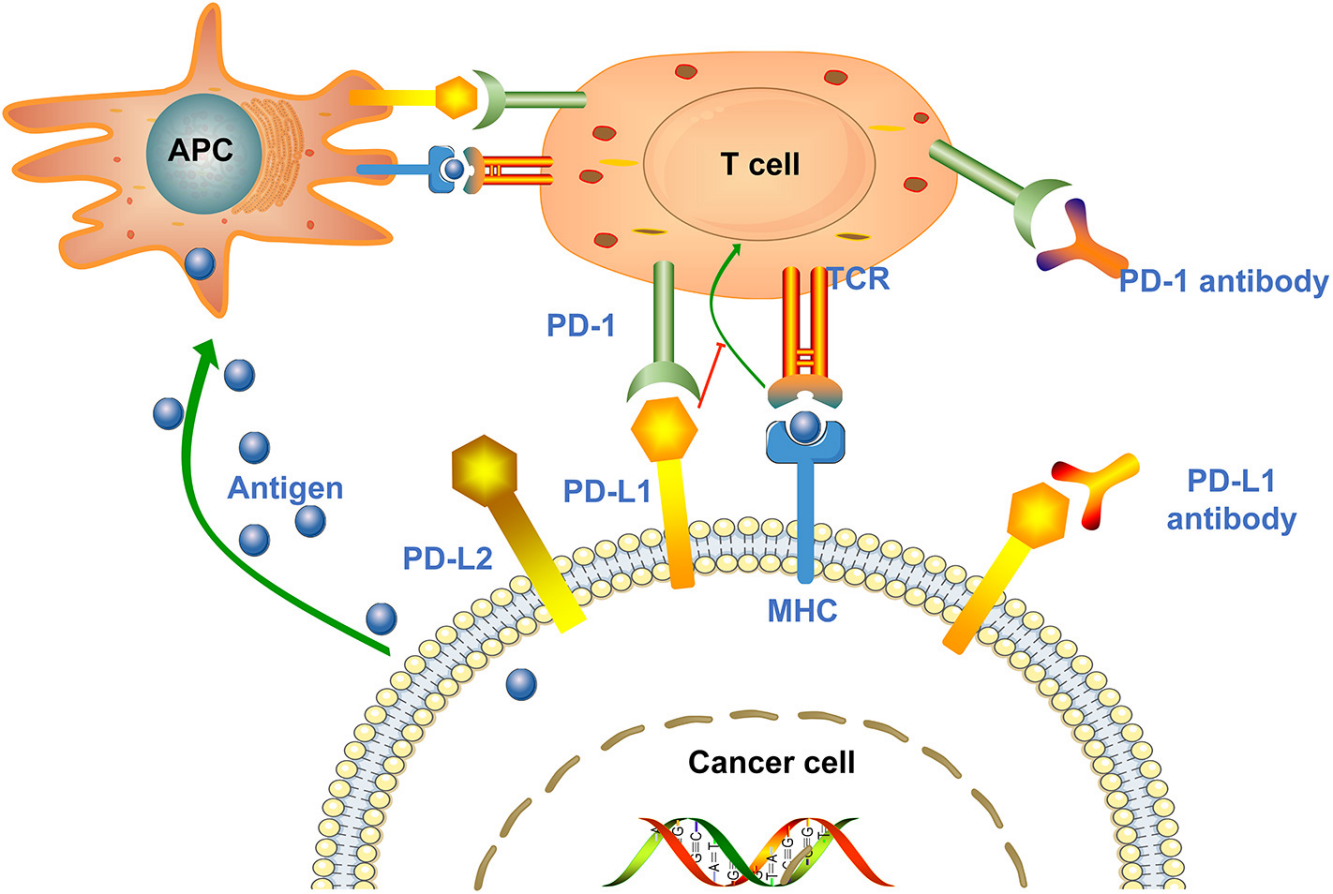
Mechanisms of cancer cell mediated immune escape. Antigen presenting cells (APCs) absorb antigens released by cancer cells and present them to T cells to promote T cells activation and high expression of PD-1. Upon T cell activation, the PD-1 receptor binds to PD-L1/PD-L2 expressed on the surface of cancer cells and suppresses the immune response. In addition, tumor cells can also present antigens directly to activated T cells in the context of MHC. Anti-PD-1/PD-L1 antibodies can block the above process and enhance the body's immune response.

PD-1, also known as CD279, is a type I transmembrane protein of the immunoglobulin superfamily (27). As a transmembrane protein, PD-1 inducibly expressed on the surface of activated T cells, B cells, NKT cells and antigen presenting cells (APC) (15, 28). PD-1 interacts with two major ligands, PD-L1 and PD-L2, resulting in disruption of intracellular signaling and down-regulation of effector T cell function (18, 29). The binding affinity of PD-1 and PD-L1 is three times than of PD-1 and PD-L2 (30). Studies showed that PD-1 is expressed in multiple type of cells, including T cells, B cells, dendritic cells, monocytes as well as tumor-infiltrating lymphocytes (TILs), while PD-L1 is expressed in cancer cells and APC (31, 32). PD-L1 expression is mainly affected by Toll-like receptors (TLRs) (33, 34). TLR-mediated PD-L1 regulation is dependent on activation of MEK/ERK kinase, which enhances PD-L1 messenger RNA (mRNA) transcription by nuclear factor kappa B (35). PD-L1 interacts with PD-1 expressed on T cells, leading to the negative regulation of effector T cell activation, thereby causing cancer cells to secrete the proinflammatory cytokines, such as TNF-α, IL-2, and IFN-γ, and become more aggressive (30). IFN-γ receptors 1 and 2 are also involved in the regulation of PD-L1 expression, primarily through JAK/STAT-mediated IRF-1 activation (35). In addition, other immunosuppressive cells in the tumor microenvironment (TME), such as regulatory T cells, tumor-associated macrophages and myeloid-derived suppressor cells, also express PD-1 to maintain a highly immunosuppressive microenvironment (Figure 1) (36, 37).

## Adverse Effects Based on PD-1/PD-L1 Blockade for NSCLC Therapy

To date, several anti-PD-1/PD-L1 immune checkpoint agents have been approved for the treatment of NSCLC, including two anti-PD-1 drugs pembrolizumab and nivolumab, as well as three anti-PD-L1 drugs atezolizumab, durvalumab and avelumab (38, 39). Blocking of PD-1/PD-L1 immune checkpoint leads to the development of new toxicities by reactivation of the immune system, also known as irAEs (26). These irAEs may affect multiple organ systems and tissues, with clinical manifestations of autoimmune-like/inflammatory side effect that may cause damage to the skin, lungs, gastrointestinal tract, liver, endocrine glands, and skeletal muscle (12). In addition, the most common treatment-related adverse events (TRAEs) include fatigue, fever/chillness and infusion reactions (9). Furthermore, rare and serious TRAEs have been reported, including immune-related encephalitis (40), myasthenia gravis (41), acute renal failure/interstitial nephritis (42), and myocarditis (43). Here, we list the TRAEs caused by PD-1 and PD-L1 inhibitors in the treatment of NSCLC in Tables 1, 2, respectively, both monotherapy and combination therapy are included.

**Table 1:** Adverse effects base on anti-PD-1 antibodies.

**Agent**	**Phase**	**ClinicalTrials.gov**	**No. of patients**	**Therapy schedule**	**TRAEs (Any grade)**	**Treatment-related serious AEs (grade 3–5)**	**References**
Pembrolizumab	I	NCT01295827	495	2 or 10 mg/kg, Q3W or 10 mg/kg, Q2W	Total: 70.9%, *n* = 351 Fatigue (19%, *n* = 96) Pruritus (11%, *n* = 53) Decreased appetite (11%, *n* = 52) Rash (10%, *n* = 48) Arthralgia (9%, *n* = 45) Diarrhea (8%, *n* = 40) Nausea (8%, *n* = 37) Hypothyroidism (7%, *n* = 34)	Total: 9.5%, *n* = 47 Decreased appetite (1%, *n* = 5) Asthenia (1%, *n* = 5) Dyspnea (4%, *n* = 19) Pneumonitis (2%, *n* = 9)	(24)
			101	2 or 10 mg/kg, Q3W or Q2W	Total: 85%, *n* = 86 Fatigue (28%, *n* = 28) Pruritus (15%, *n* = 15) Hypothyroidism (14%, *n* = 14) Rash (14%, *n* = 14) Arthralgia (12%, *n* = 12) Nausea (12%, *n* = 12) Dyspnea (9%, *n* = 9) Diarrhea (8%, *n* = 8)	Total: 12%, *n* = 12 Hypertension (1%, *n* = 1) Colitis (1%, *n* = 1) Dehydration (1%, n=1) Dyspnea (1%, *n* = 1) Pneumonitis (1%, *n* = 1)	(44)
Pembrolizumab	III	NCT02220894	636	200 mg, Q3W	Total: 63%, *n* = 399 Hypothyroidism (11%, *n* = 69) Fatigue (8%, *n* = 50) Pruritus (7%, *n* = 46) Rash (7%, n=46) Alanine aminotransferase increased (7%, *n* = 45) Pneumonitis (7%, *n* = 43) Decreased appetite (6%, *n* = 40) Hyperthyroidism (6%, *n* = 37)	Total: 18%, *n* = 113 Pneumonitis (3%, *n* = 20) Alanine aminotransferase increased (1%, *n* = 9) Hypothyroidism (<1%, *n* = 1) Fatigue (<1%, *n* = 3)	(45)
Pembrolizumab	II/III	NCT01905657	691	2 or 10 mg/kg, Q3W	Total: 64%, *n* = 441 Fatigue (28%, *n* = 95) Decreased appetite (24%, *n* = 79) Nausea (20%, *n* = 68) Rash (22%%, *n* = 73) Diarrhea (13%, *n* = 46) Asthenia (12%, *n* = 39) Stomatitis (6%, *n* = 20) Anemia (7%, *n* = 24)	Total: 14%, *n* = 98 Fatigue (3%, *n* = 10) Decreased appetite (<2%, *n* = 4) Nausea (<2%, *n* = 3) Diarrhea (1%, *n* = 2)	(46)
Pembrolizumab	III	NCT02142738	154	200 mg, Q3W	Total: 73%, *n* = 113 Diarrhea (14%, *n* = 22) Pyrexia (10%, n=16) Fatigue (10%, *n* = 16) Nausea (10%, *n* = 15) Decreased appetite (9%, *n* = 14) Anemia (5%, *n* = 8) Constipation (4%, *n* = 6) Vomiting (3%, *n* = 4)	Total: 27%, *n* = 41 Diarrhea (4%, *n* = 6) Anemia (2%, *n* = 3) Fatigue (1%, *n* = 2)	(47)
Pembrolizumab + pemetrexed + carboplatin	II	NCT02039674	59	Pembrolizumab 200 mg, Q3W plus chemotherapy	Total: 93%, *n* = 55 Fatigue (61%, *n* = 36) Nausea (56%, *n* = 33) Anemia (20%, *n* = 12) Vomiting (25%, *n* = 15) Rash (25%, *n* = 15) Decreased appetite (19%, *n* = 11) Diarrhea (20%, *n* = 12) Increased aspartate (17%, *n* = 10)	Total: 39%, *n* = 23 Fatigue (3%, *n* = 2) Acute kidney injury (3%, *n* = 1) Anemia (12%, *n* = 7) Neutropenia (3%, *n* = 2) Decreased neutrophil count (3%, *n* = 2)	(48)
Pembrolizumab + pemetrexed + platinum-based drug	III	NCT02578680	405	Pembrolizumab 200 mg, Q3W plus chemotherapy	Total: 99%, *n* = 404 Nausea (56%, *n* = 225) Fatigue (46%, *n* = 187) Anemia (41%, *n* = 165) Constipation (35%, *n* = 141)	Total: 67%, *n* = 272 Anemia (16%, *n* = 66) Neutropenia (15.8%, *n* = 64) Thrombocytopenia (8%, *n* = 32) Asthenia (6%, *n* = 25)	(49)
					Diarrhea (31%, *n* = 125) Decreased appetite (28%, *n* = 114) Neutropenia (27%, *n* = 110) Vomiting (24%, *n* = 98)	Fatigue (6%, *n* = 23) Diarrhea (5%, *n* = 21) Nausea (4%, *n* = 14)	
Pembrolizumab + carboplatin + paclitaxel or nab-paclitaxel	III	NCT02775435	278	Pembrolizumab 200 mg, Q3W plus chemotherapy	Total: 98%, *n* = 273 Anemia (53%, *n* = 148) Alopecia (46%, *n* = 128) Neutropenia (38%, *n* = 105) Nausea (36%, *n* = 99) Thrombocytopenia (31%, *n* = 85) Diarrhea (30%, *n* = 83) Decreased appetite (25%, *n* = 68) Constipation (23%, *n* = 64)	Total: 70%, *n* = 194 Anemia (16%, *n* = 43) Neutropenia (23%, n=63) Thrombocytopenia (7%, *n* = 19) Diarrhea (4%, *n* = 11) Decreased appetite (2%, *n* = 6)	(50)
Nivolumab	III	NCT01642004	131	3 mg/kg, Q2W	Total: 58%, *n* = 76 Fatigue (16%, *n* = 21) Decreased appetite (11%, *n* = 14) Asthenia (10%, *n* = 13) Nausea (9%, *n* = 12) Diarrhea (8%, *n* = 10) Arthralgia (5%, *n* = 7) Pneumonitis (5%, *n* = 6) Pyrexia (5%, *n* = 6)	Total: 7%, *n* = 9 Fatigue (1%, *n* = 1) Decreased appetite (1%, *n* = 1) Leukopenia (1%, *n* = 1)	(51)
Nivolumab	III	NCT01673867	287	3 mg/kg, Q2W	Total: 69%, *n* = 199 Fatigue (16%, *n* = 46) Nausea (12%, *n* = 34) Decreased appetite (10%, *n* = 30) Asthenia (10%, *n* = 29) Diarrhea (8%, *n* = 22) Peripheral edema (3%, *n* = 8) Myalgia (2%, *n* = 7) Anemia (2%, *n* = 6)	Total: 10%, *n* = 30 Fatigue (1%, *n* = 3) Nausea (1%, *n* = 2) Asthenia (<1%, *n* = 1) Diarrhea (<1%, *n* = 2)	(52)
Nivolumab	II	NCT01721759	117	3 mg/kg, Q2W	Total: 74%, *n* = 87 Fatigue (33%, *n* = 38) Asthenia (12%, *n* = 14) Nausea (15%, *n* = 18) Diarrhea (10%, *n* = 12) Decreased appetite (19%, *n* = 22) Rash (11%, *n* = 13) Anemia (6%, *n* = 7) Pneumonitis (5%, *n* = 6)	Total: 17%, *n* = 20 Fatigue (4%, *n* = 5) Diarrhea (3%, *n* = 3) Rash (1%, *n* = 1) Pneumonitis (1%, *n* = 1) Anemia (1%, *n* = 1)	(53)
Nivolumab	I	NCT01454102	52	3 mg/kg, Q2W	Total: 71%, *n* = 37 Fatigue (29%, *n* = 15) Rash (19%, *n* = 10) Nausea (14%, *n* = 7) Diarrhea (12%, *n* = 6) Pruritus (12%, *n* = 6) Arthralgia (6%, *n* = 3) Constipation (6%, *n* = 3) Hypothyroidism (6%, *n* = 3)	Total: 19%, *n* = 10 Rash (4%, *n* = 2) Diarrhea (2%, *n* = 1) Pneumonitis (2%, *n* = 1)	(54)
Nivolumab + Ipilimumab	I	NCT01454102	38	Nivolumab 3 mg/kg, Q2W + ipilimumab 1 mg/kg, Q12W	Total: 82%, *n* = 31 Pruritus (24%, *n* = 9) Diarrhea (21%, *n* = 8) Nausea (16%, *n* = 6) Fatigue (16%, *n* = 6) Increased amylase (16%, *n* = 6) Maculopapular rash (13%, *n* = 5) Pyrexia (13%, *n* = 5) Rash (16%, *n* = 6)	Total: 37%, *n* = 14 Increased lipase (8%, *n* = 3) Pneumonitis (5%, *n* = 2) Diarrhea (3%, *n* = 1) Fatigue (3%, *n* = 1) Rash (3%, *n* = 1)	(55)
			39	Nivolumab 3 mg/kg Q2W + ipilimumab 1 mg/kg Q6W	Total: 72%, *n* = 28 Pruritus (13%, *n* = 5) Diarrhea (21%, *n* = 8) Nausea (16%, *n* = 6) Fatigue (23%, *n* = 9) Maculopapular rash (10%, *n* = 4) Pyrexia (5%, *n* = 2) Rash (10%, *n* = 4) Decreased appetite (13%, *n* = 5)	Total: 33%, *n* = 13 Adrenal insufficiency (5%, *n* = 2) Colitis (5%, *n* = 2) Nausea (3%, *n* = 1) Fatigue (3%, *n* = 1) Maculopapular rash (3%, *n* = 1)	(55)
Nivolumab +cisplatin + gemcitabine or paclitaxel	I	NCT01454102	56	5 or 10 mg/kg, Q3W + chemotherapy	Total: 95%, *n* = 53 Fatigue (71%, *n* = 40) Nausea (46%, *n* = 26) Decreased appetite (36%, *n* = 20) Alopecia (30%, *n* = 17) Anemia (27%, *n* = 15) Rash (27%, *n* = 15) Diarrhea (21%, *n* = 12)	Total: 45%, *n* = 25 Pneumonitis (7%, *n* = 4) Fatigue (5%, *n* = 3) Acute renal failure (5%, *n* = 3) Anemia (4%, *n* = 2) Neutropenia (4%, *n* = 2)	(56)
Nivolumab + ALT-803	Ib	NCT02523469	21	Nivolumab 3 mg / kg, Q2W + ALT-803 6,10,15 or 20μg/ kg, Q1W	Injection-site reaction (90%, *n* = 19) Flu-like symptoms (71%, *n* = 15) Fever (67%, *n* = 10) Chills (29%, *n* = 6) Nausea (38%, *n* = 8) Pain (33%, *n* = 7) Dizziness (24%, *n* = 5)	Fatigue (10%, *n* = 2) Lymphocytopenia (10%, *n* = 2) Fever (5%, *n* = 1) Anemia (5%, *n* = 1) Abdominal pain (5%, *n* = 1)	(57)

**Table 2:** Adverse effects base on anti-PD-L1 antibodies.

**Agent**	**Phase**	**ClinicalTrials.gov**	**No. of patients receiving** **anti-PD-L1 agent**	**Therapy schedule**	**TRAEs (Any grade)**	**Treatment-related serious AEs (grade 3–5)**	**References**
Atezolizumab	II	NCT02031458	659	1,200 mg, Q3W	Total: 65%, *n* = 429 Fatigue (19%, *n* = 122) Diarrhea (11%, *n* = 71) Nausea (11%, *n* = 73) Pruritus (10%, *n* = 65) Pyrexia (8%, *n* = 54) Decreased appetite (8%, *n* = 53) Asthenia (8%, *n* = 50) Rash (8%, *n* = 50)	Total: 12%, *n* = 82 Fatigue (1%, *n* = 7) Nausea (1%, *n* = 4) Asthenia (1%, *n* = 3) Rash (1%, *n* = 9)	(58)
Atezolizumab	I	NCT01375842	89	1-20 mg/kg or 1,200 mg, Q3W	Total: 76%, *n* = 68 Fatigue (20%, *n* = 18) Nausea (16%, *n* = 14) Decreased appetite (14%, *n* = 12) Asthenia (10%, *n* = 9) Rash (9%, *n* = 8) Dyspnea (8%, *n* = 7) Diarrhea (8%, *n* = 7) Headache (7%, *n* = 6)	Total: 11%, *n* = 10 Fatigue (2%, *n* = 2) Dyspnea (2%, *n* = 2) Nausea (1%, *n* = 1) Vomiting (1%, *n* = 1)	(59)
Atezolizumab	III	NCT02008227	609	1,200 mg, Q3W	Total: 94%, *n* = 573 Fatigue (27%, *n* = 163) Decreased appetite (24%, *n* = 143) Cough (23%, *n* = 141) Nausea (18%, *n* = 108) Diarrhea (15%, *n* = 94) Asthenia (19%, *n* = 116) Dyspnea (19%, *n* = 118) Anemia (12%, *n* = 70)	Total: 37%, *n* = 227 Fatigue (3%, *n* = 17) Dyspnea (3%, *n* = 15) Anemia (2%, *n* = 14) Asthenia (1%, *n* = 8) Back pain (1%, *n* = 7)	(60)
Atezolizumab	II	NCT01846416	137	1,200 mg, Q3W	Total: 70%, *n* = 96 Fatigue (27%, *n* = 37) Decreased appetite (15%, *n* = 21) Nausea (15%, *n* = 20) Diarrhea (10%, *n* = 13) Pyrexia (8%, *n* = 11) Pruritus (7%, *n* = 10) Arthralgia (7%, *n* = 9) Rash (7%, *n* = 9)	Not mentioned	(61)
Atezolizumab + carboplatin + paclitaxel or pemetrexed or nab-paclitaxel	I	NCT01633970	76	Atezolizumab 15 mg/kg, Q3W + chemotherapy	Not mentioned	Total: 72%, *n* = 55 Neutropenia (38%, *n* = 29) Anemia (21%, *n* = 16) Fatigue (11%, *n* = 8) Thrombocytopenia (8%, *n* = 6) Febrile neutropenia (7%, *n* = 5) Neutrophil count decreased (7%, *n* = 5) Platelet count decreased (5%, *n* = 4) Dehydration (5%, *n* = 4)	(62)
Atezolizumab + bevacizumab + carboplatin +paclitaxel	III	NCT02366143	393	Atezolizumab 1,200 mg, Q3W + chemotherapy	Total: 94.4%, *n* = 371 Alopecia (47%, *n* = 183) Peripheral neuropathy (36%, *n* = 141) Nausea (30%, *n* = 119) Fatigue (22%, *n* = 88) Decreased appetite (20%, *n* = 77) Anemia (18%, *n* = 70) Diarrhea (18%, *n* = 70) Constipation (17%, *n* = 65)	Total: 59%, *n* = 230 Neutropenia (14%, *n* = 54) Decreased neutrophil count (9%, *n* = 34) Febrile neutropenia (9%, *n* = 36) Hypertension (6%, *n* = 25) Anemia (6%, *n* = 24) Decreased platelet count (5%, *n* = 20)	(63)
Durvalumab	II	NCT02087423	444	10 mg/kg, Q2W	Total: 58%, *n* = 256 Fatigue (11%, *n* = 50) Hypothyroidism (8%, *n* = 36) Asthenia (7%, *n* = 31) Nausea (6%, *n* = 28) Pruritus (6%, *n* = 28) Diarrhea (6%, *n* = 27) Vomiting (3%, *n* = 14) Anemia (2%, *n* = 9)	Total: 9%, *n* = 40 Fatigue (<1%, *n* = 2) Vomiting (<1%, *n* = 2) Pneumonitis (1%, *n* = 4) Gamma-glutamyltransferase increased (1%, *n* = 4)	(64)
Durvalumab	III	NCT02125461	475	10 mg/kg, Q2W	Total: 67.8%, *n* = 322 Fatigue (13%, *n* = 62) Hypothyroidism (11%, *n* = 65) Diarrhea (10%, *n* = 46) Pneumonitis (9%, *n* = 43) Rash (8%, *n* = 37) Pruritus (7%, *n* = 33) Hyperthyroidism (6%, *n* = 30) Asthenia (6%, *n* = 28)	Total: 12%, *n* = 56 Pneumonitis (1%, *n* = 6) Asthenia (<1%, *n* = 3) Dyspnea (<1%, *n* = 3)	(65)
Durvalumab +Tremelimumab	I	NCT02000947	102	Durvalumab 10-20 mg/kg, Q4W + Tremelimumab 1-3 mg/kg, Q12W	Total: 80%, *n* = 82 Diarrhea (32%, *n* = 33) Colitis (12%, *n* = 12) Pruritus (21%, *n* = 21) Rash (15%, *n* = 15) Hypothyroidism (10%, *n* = 10) Pneumonitis (5%, *n* = 5) Rash maculopapular (4%, *n* = 4)	Total: 42%, *n* = 43 Diarrhea (11%, *n* = 11) Colitis (9%, *n* = 9) Pneumonitis (4%, *n* = 4) Enteritis (1%, *n* = 1) Hypothyroidism (1%, *n* = 1)	(66)
Avelumab	I	NCT01772004	184	10 mg/kg, Q2W	Total: 77%, *n* = 142 Fatigue (25%, *n* = 46) Infusion-related reaction (19%, *n* = 34) Nausea (13%, *n* = 23) Decreased appetite (7%, *n* = 13) Diarrhea (7%, *n* = 13) Chills (7%, *n* = 12) Hypothyroidism (6%, *n* = 11)	Total: 13%, *n* = 23 Elevated lipase (2%, *n* = 3) Infusion-related reaction (1%, *n* = 2) Dyspnea (1%, *n* = 2) Elevated amylase (1%, *n* = 1) Autoimmune neutropenia (1%, *n* = 1)	(67)
Avelumab	III	NCT02395172	393	10 mg/kg, Q2W	Total: 64%, *n* = 251 Infusion-related reaction (15%, *n* = 59) Decreased appetite (9%, *n* = 34) Fatigue (7%, *n* = 29) Asthenia (7%, *n* = 29) Diarrhea (6%, *n* = *n* = 24) Nausea (5%, *n* = 20) Myalgia (2%, *n* = *n* = 6) Mucosal inflammation (1%, *n* = 2)	Total: 12%, *n* = 47 Infusion-related reaction (1%, *n* = 5) Lipase increased (1%, *n* = 3) Asthenia (<1%, *n* = 1) Fatigue (<1%, *n* = 1)	(68)

## Comparison of the Toxicity Spectrum Between PD-1 and PD-L1 Inhibitors in the Treatment of NSCLC

At present, although various PD-1 and PD-L1 ICIs have shown activity in NSCLC, it is meaningful to analyze and compare the differences in their toxicity profiles (69). According to the results of a systematic meta-analysis by Pillai et al., there was no significant difference in the overall incidence of AEs between the PD-1 treatment group (*n* = 3284) and the PD-L1 treatment group (*n* = 2460) (69–71). However, any grade of irAEs in the PD-1 treatment group was slightly higher than the PD-L1 treatment group (16 vs. 11%; *p* = 0.07) (69). The most common AE of PD-1 and PD-L1 inhibitors is fatigue (19 vs. 21%, *p* = 0.4), while the most common irAE is hypothyroidism (6.7 vs. 4.2%; *p* = 0.07) (69). It was worth noting that in patients receiving PD-1 inhibitors, the incidence of pneumonitis was significantly higher than in the PD-L1 agents treatment group (4 vs. 2%; *P* = 0.01) (69, 70). Therefore, clinicians should be more alert to lung inflammation in NSCLC patients receiving PD-1 blockade therapy (69).

At present, there is no systematic study to analyze the differences in the toxic and side effects of PD-1/PD-L1 inhibitors alone or in combination with other therapies for NSCLC. However, the current clinical trial data seems to indicate that the overall incidence of AEs of PD-1/PD-L1 inhibitor monotherapy is lower than that of combination therapy. For example, any grade of TRAEs that occurred with pembrolizumab monotherapy was 70.9% (24), while pembrolizumab combined with chemotherapy showed a higher incidence of TRAEs (98.2%) (50). Several other clinical trials of PD-1/PD-L1 inhibitors that have been approved for the treatment of NSCLC also showed the same trend (54, 56).

## Management of Organ-Specific Toxicities Caused by Anti-PD-1/PD-L1 Treatment

### Skin-Related Adverse Events

Rash and pruritus are the most common skin irAEs that occur in NSCLC patients receiving anti-PD-1/PD-L1 immune checkpoint treatment (12). Skin-related irAEs usually occur after the second cycle of the patient's clinical course (72, 73). Other dermatological lesions include vitiligo, skin capillary hyperplasia (CCEP), lichenoid and bullous pemphigoid (74). Despite frequent reports of immune-related skin AEs, the incidence of skin AEs of grade III or higher is low, and life-threatening AEs are only occasionally reported, but still deserve attention (74). For PD-1/PD-L1 monotherapy, the incidence of treatment-related skin AEs of any grade is ~7–31%, and the incidence of grade III or higher AEs is lower. Existing clinical trial data showed that the incidence of skin-related AEs of anti-PD-1 monotherapy was slightly higher than that of anti-PD-L1 monotherapy (11–31 vs. 7–19%) (24, 53, 67, 75). In addition, the emergence of skin toxicity caused by pembrolizumab seems to be more frequent than other anti-PD-1/PD-L1 agents (24, 67, 75). However, there was no significant difference in the incidence of skin-related AEs between anti-PD-1/PD-L1 monotherapy and combination therapy (24, 47). Recent studies have shown that patients with complete/partial remission have a higher incidence of skin adverse reactions than patients with stable/progressive disease, suggesting that skin AEs may be a positive prognostic factor for patients, but more prospective studies are still needed to further verify this kind of association (76, 77). However, a basic skin examination is necessary for patients using ICIs, especially those with previous inflammatory skin diseases. Standard dermatological assessments include skin biopsies, kidney and liver function tests, serum tryptase as well as immunoglobulin E levels (74).

For mild (grade I–II) maculopapular patients, it may be managed successfully with moderate potency topical steroids to affected areas and/or oral prednisone 0.5–1 mg/kg/day (78). For grade III–IV maculopapular, immunotherapy may be temporarily held and patients should be treated with high potency topical steroids to affected areas and oral prednisone 0.5–1 mg/kg/day (increase dose up to 2 mg/kg/day if no improvement) (79). In addition, topical emollients, oral antihistamines and lidocaine patches are effective for pruritus. For patients with severe pruritus, the GABA agonists (gabapentin, pregabalin) are useful, and aprepitant or omalizumab can be used in refractory cases (79).

### Respiratory System Related Adverse Events

Anti-PD-1/PD-L1 immunotherapy also frequently occurs respiratory system-related AEs, especially for patients with lung cancer, the incidence of such AEs seems to be higher (69). Among them, immune-related pneumonia is the most common. Pneumonia is defined as focal or diffuse inflammation of the lung parenchyma, including pulmonary sarcoidosis and organizing inflammatory pneumonitis (80). Once pneumonia occurs, it may endanger the life of the patient, so active interventions should be taken (12, 80). The incidence of pneumonia is generally 7.4–24.3 months after the start of treatment. The clinical symptoms are mainly dry cough, dyspnea, fever, and chest pain (12, 81). It is worth noting that the combination of ICIs and other drugs at risk of pneumonia will increase the incidence of pneumonia (82). Chen et al. (83) reported an unpredictable but relatively severe radiation recall pneumonitis (RRP), which was induced by anti-PD-1 inhibitor camrelizumab 2 years after radiotherapy. This indicated that previous radiotherapy combined with subsequent anti-PD-1 immunotherapy may result in overlapping damage to lung (83). Moreover, patients with other underlying lung diseases, such as COPD and pulmonary fibrosis, should be more alert to the occurrence of pneumonia (84, 85).

Chest CT is a key method for diagnosing pneumonia. The imaging features are ground-glass lesions and/or disseminated nodular infiltrates (12, 86). According to the management of the latest NCCN guidelines, any level of immune-related pneumonia should hold immunotherapy, and patients with severe pneumonia should permanently discontinue immunotherapy. Patients with mild (grade I) pneumonia need to re-evaluate arterial oxygen saturation (both resting and active) and repeat chest CT in 4 weeks or as clinically indicated for worsening symptoms (78, 87). For grade II or higher pneumonia should first rule out bacterial infections, such as nasal swab for potential viral pathogens, sputum culture, blood culture, and urine antigen test to detect pneumococcus and legionella (87). Additionally, bronchoscopy and bronchoalveolar lavage are necessary. If the infection cannot be completely ruled out, empiric antibiotics can be used. Management is guided by clinical symptoms, such that grade II pneumonia patients can be taken orally or intravenously prednisone/methylprednisolone 1–2 mg/kg/day (86, 87). Severe cases require hospitalization and intravenous methylprednisolone 1–2 mg/kg/day. Other forms of immunosuppression may be considered, such as infliximab, mycophenolate mofetil or intravenous immunoglobulin, if corticosteroids remain ineffective after 48 h of treatment (86, 87).

### Digestive System Related Adverse Events

Colitis and diarrhea are the most common gastrointestinal toxicity during the treatment of anti-PD-1/PD-L1 immunotherapy (24). Other gastrointestinal adverse reactions include decreased appetite, nausea, vomiting, constipation (24). Colitis clinically involves clinical or imaging evidence of abdominal pain symptoms and colon inflammation, while diarrhea refers to an increase in stool frequency (72). In immune checkpoint blocking therapy, the incidence of gastrointestinal AEs with anti-CTLA-4 treatment is higher than that with anti-PD-1/PD-L1 therapy (72). Moreover, anti-PD-1/PD-L1 agents combined with chemotherapy drugs will increase the incidence of gastrointestinal AEs (any grade) (23, 56). In general, the incidence of grade III–IV colitis/diarrhea is about 5% and life-threatening cases are rarely reported (12). In clinical management of immune-related colitis and diarrhea AEs, stool evaluation should be performed to rule out any possible bacterial, viral pathogen, and parasitic infections (88). For mild diarrhea or colitis, it is useful to oral loperamide or diphenoxylate/atropine for 2–3 days and hydration (78, 88). Moderate or severe colitis/diarrhea should hold immunotherapy. Patients with grade 3 may consider re-use of anti-PD-1/PD-L1 therapy after toxicity has been relieved, but patients with grade IV should permanently discontinue immunotherapy (78). Patients with grade IV may be successfully managed by using prednisone/methylprednisolone (1–2 mg/kg/day). If the symptoms do not improve, consider adding infliximab or vedolizumab within 2 weeks. Severe cases should be hospitalized to provide supportive treatment (78).

### Hepatic Toxicities

Among NSCLC patients receiving anti-PD-1/PD-L1 immunotherapy, the incidence of immune-related hepatitis is approximately 5%, while the incidence of severe hepatitis (grade III-IV) is <2% (89). The median time to onset is usually 6–14 weeks from the first taking of anti-PD-1/PD-L1 drugs, but may occur within a few months after starting treatment or even stopping treatment (89). Any asymptomatic elevations in alanine aminotransferase (ALT) or aspartate aminotransferase (AST) enzymes levels should consider immune-related hepatitis (78, 90). Some patients occasionally observe elevated levels of bilirubin, usually without obvious symptoms. In addition, liver biopsy is the gold standard for diagnosing and evaluating the degree of autoimmune hepatitis and liver injury (90). The clinical symptoms of immune-mediated hepatitis include hepatomegaly, portal and periportal inflammation, lymphadenomegaly, and infiltrating eosinophils, lymphocytes as well as plasma cells (90). Before treatment of immune-related hepatitis, viral etiology (hepatitis A, hepatitis B or C, and emergency hepatitis E virus), disease-related liver dysfunction, and other drug-induced transaminase elevations should be excluded. Ultrasound or magnetic resonance cholangiopancreatography can be considered to rule out liver metastases or gallstones of cancer (78). For mild to moderate hepatitis (grade I–II), immunotherapy can be continued or suspended according to the patient's condition, and liver function tests (LFTs) are closely monitored. Patients with grade III–IV hepatitis should permanently discontinue immunotherapy and use prednisone/methylprednisolone 1–2 mg/kg/day. If the steroid treatment does not improve after 3 days, consider adding an additional immunosuppressant mycophenolates, but should not use infliximab as its potential hepatotoxicity (78).

### Endocrine System Related Adverse Events

The endocrine system contains many important organs of the human body, such as hypothalamus, pituitary, thyroid, adrenal glands, and pancreas. The endocrine toxicity caused by PD-1/PD-L1 ICIs may affect any axis (12). Hypophysitis, thyroiditis, hypothyroidism, hyperthyroidism, and adrenal insufficiency are common immune-related endocrine diseases (44). Among patients with NSCLC, hypothyroidism is the most common endocrine toxicity, with an incidence of 5–15% (44). Since the clinical symptoms of immune endocrine disease are non-specific, such as fatigue, headache, and nausea. Cancer patients are often accompanied by such symptoms. Therefore, the diagnosis of immune-mediated endocrine toxicity is clinically challenging (12). Clinically, endocrine diseases such as central hypothyroidism and pituitary inflammation are diagnosed by evaluating biochemical indicators such as morning cortisol, ACTH (adreno-cortico-tropic-hormone), FSH (follicle-stimulating hormone), LH (luteinizing hormone), TSH (thyroid stimulating hormone), free T4, and DHEA-S (91). For patients with hypothyroidism, the thyroid hormone replacement therapy may be useful, and closely monitor the level of TSH is necessary (every 4–6 weeks) (78). If TSH > 10, levothyroxine should be used to make TSH reach the reference range or age-appropriate range. Patients with hyperthyroidism can be treated with standard antithyroid drugs. In addition, pituitary inflammation with obvious symptoms can be considered with prednisone/methylprednisolone 1–2 mg/kg/day for treatment (78). Primary adrenal insufficiency occurs less frequently in irAEs related to PD-1/PD-L1 blockade therapy, but in rare cases an adrenal crisis may occur (91). It should hold the immunotherapy and perform intravenous corticosteroid as well as supplement aggressive fluid and electrolyte when such AEs occur (91). Most endocrine-related toxicity is effective through hormone replacement therapy, without holding PD-1/PD-L1 immune checkpoint treatment.

### Skeletal Muscle System Related Adverse Events

Some tumor patients receiving anti-immunity checkpoint treatment will also have skeletal muscle system-related AEs, but musculoskeletal symptoms are also present in the tumor patients themselves, therefore more attention should be paid to distinguishing (81). Overall, the majority of immune-related muscle AEs in patients with NSCLC are mild (grade I-II). The diagnosis of inflammatory arthritis is mainly by evaluating the degree of joint involvement, X-ray and joint ultrasound (78). Moreover, it is necessary to check the creation kinase/aldolase and troponin levels. NSCLC patients have the most reported immune-related muscle adverse reaction is myalgia (43). Patients with mild pain can continue immunotherapy and continuously monitor serial aldolase/creatine kinase levels, but moderate or severe pain should hold immunotherapy, using prednisone 1–2 mg/kg/day for treatment and considering muscle biopsy (72).

## Management of Other Common Adverse Events

### Fatigue

Fatigue widely occurs in patients with NSCLC who are treated with PD-1/PD-L1 immune checkpoint blockade (12). Overall, for NSCLC patients receiving anti-PD-1/PD-L1 monotherapy or combination therapy, ~6–71% of patients reported treatment-related fatigue (any grade), but the incidence of grade III/IV is low (<5%) (24, 45, 56). Compared with anti-PD-1/PD-L1 monotherapy, PD-1/PD-L1 ICIs combined with other therapies (chemotherapy, targeted therapy, anti-CTLA-4 therapy) significantly increased the incidence of fatigue side effects (6–33 vs. 13–71%) (47, 54, 75). However, it is worth noting that fatigue symptoms are sometimes caused by immune-related endocrine toxicity. For example, early symptoms of hypothyroidism can also cause fatigue (81). Therefore, the treatment of fatigue should consultation based on abnormalities, and the use of low-dose steroids is allowed (78). In addition, moderate physical activity and psychosocial intervention can also help relieve fatigue symptoms (72). For severe fatigue, consideration should be given to whether tumor disease progression or other medical diseases occur (78).

### Pyrexia/Chills and Infusion Reactions

Anti-PD-1/PD-L1 immune checkpoint therapy may cause cytokine release and non-specific over-activation of the immune system, which may lead to symptoms of pyrexia, chill and infusion reactions in patients (81). Approximately 5–18% of patients with NSCLC develop immune-related pyrexia during treatment. It can be managed by using antipyretics, such as acetaminophen or non-steroidal anti-inflammatory drugs (78). For grade I–II infusion reactions, it can resume infusion or reduce the infusion rate after the symptoms disappear, and consider premedication with acetaminophen, famotidine, and diphenhydramine with future infusions. For grade III infusion reactions, the immunotherapy should be permanently discontinued, and intravenous antihistamine or corticosteroid drugs are required (74, 78).

## Management of Rare But Serious Adverse Events

### Immune-Related Encephalitis

Immune-related encephalitis is a rare and poorly understood irAE, with an incidence of <1% in cancer patients undergoing immune checkpoint blockade therapy, but it may be fatal (92). Therefore, it is necessary to increase its awareness for effective management. A multicenter cohort retrospectively analyzed the clinical, biological, and radiological characteristics of nine immune-related encephalitis in NSCLC patients undergoing anti-PD-1/PD-L1 treatment (40). The most common clinical symptoms of these patients include fever, confusion, and cerebellar ataxia (40). In addition, it was found that the levels of white blood cell increased, without any bacterial and viral infection. One patient's brain MRI examination showed that the limbic system is involved, which is fatal (40). The most important management of immune-related encephalitis is early treatment with corticosteroids (prednisone 1–2 mg/kg/day). Severe cases should permanently discontinue immunotherapy (78).

### Myasthenia Gravis

The immune-related myasthenia gravis is also a rare but serious neurotoxicity caused by anti-PD-1/PD-L1 treatment (43, 91). The average onset time of the patient's symptoms appeared within 6 weeks of starting treatment (range 2–12 weeks) (93). Treatment-related reports of myasthenia gravis in NSCLC patients receiving PD-1 monoclonal antibodies seem to be more common than those receiving PD-L1 agents (41, 94, 95). A 63-year-old female patient with stage IV NSCLC adenocarcinoma, who failed conventional chemotherapy (disease progression) and subsequently used pembrolizumab, was diagnosed with myasthenia gravis after two cycles of treatment (41). The clinical symptoms are bilateral eyelid drooping, extraocular muscle paralysis, shortness of breath, and fatigue (41). Moreover, two patients with NSCLC who received nivolumab reported myasthenia gravis, and the onset time was within 2–3 cycles after the start of treatment (94, 95). Moderate and severe autoimmune myasthenia gravis should permanently discontinued immunotherapy, as well as oral pyridostigmine 30 mg TID and gradually increase to maximum of 120 mg four times a day as tolerated and based on symptoms (93). In addition, considering low-dose oral prednisone 20 mg daily and gradually increase the dose (not more than 100 mg/day) if necessary. Severe cases should use methylprednisolone 1–2 mg/kg/day and consider adding rituximab (375 mg/m^2^ weekly for 4 treatments or 500 mg/m^2^ every 2 weeks for 2 doses) if refractory to plasmapheresis or intravenous immunoglobulin (IVIG) (93).

### Acute Renal Failure/Interstitial Nephritis

The main manifestation of kidney injury is elevated serum creatinine levels, and patients usually develop acute renal failure and interstitial nephritis (96). According to reports, the possible mechanism of kidney damage induced by ICIs is that drugs or drug metabolites activate circulating T cells, which binding to carrier proteins and form drug-carrier immune complexes to obtain immunogenicity (97). When these immune complexes are presented as a local antigen to the kidney, they trigger a hypersensitivity reaction through the release of cytokines, leading to the occurrence of kidney damage (97). In NSCLC patients, a phase I study (NCT01454102) of nivolumab combined with platinum-based dual chemotherapy reported 3 cases of grade 3 acute renal failure. In addition, Koda et al. (42) reported a 67-year-old stage IV acute tubulointerstitial nephritis caused by nivolumab monotherapy in patients with NSCLC. For the management of acute renal failure/interstitial nephritis, creatinine, and urine protein levels should be closely monitored (once every 3–7 days), and prednisone 0.5–1 mg/kg/day may be useful (42). Patients with severe kidney injury should permanently discontinue immunotherapy and use prednisone/methylprednisolone 1–2 mg/kg/day. Conduct renal biopsy and nephrology consultation if necessary. Moreover, add one of the following drugs, azathioprine, cyclophosphamide, cyclosporine, infliximab, and mycophenolate, if the symptoms still not improve after treated with steroids for more than 1 week (42).

### Myocarditis

**Figure 2:**
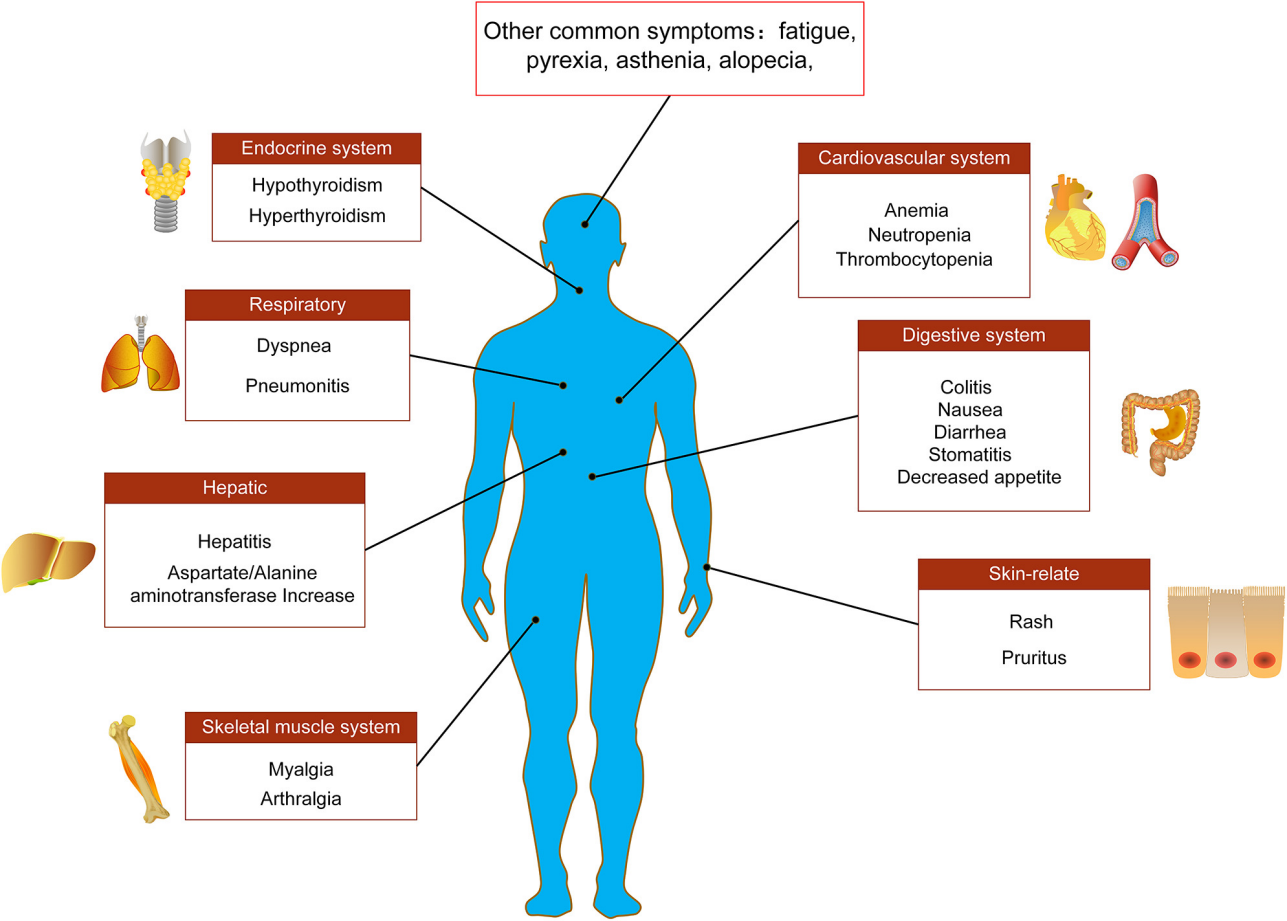
Main adverse events of PD-1/PD-L1 immunotherapy. Adverse events associated with PD-1/PD-L1 immune checkpoint inhibitors in the treatment of NSCLC involve multiple tissues and organs, including endocrine system, respiratory system, digestive system, cardiovascular system, skeletal muscle system, liver, and skin-related adverse reactions.

Immune-mediated cardiotoxicity, myocarditis, is a rare but serious side effect in NSCLC patients receiving anti-PD-1/PD-L1 immune checkpoint treatment, which needs to be recognized as soon as possible for better management (98–100). A case report showed that a 75-year-old NSCLC patient suffered a drug-induced AE of myocarditis during the ninth cycle of nivolumab treatment, and its clinical symptoms were dyspnea and acute chest pain (98). After treatment with ACE-inhibitors, β-blockers and diuretics as well as prednisolone (1 mg/kg/day), the cardiac function of patient was significantly improved (98). Similarly, Gibson et al. (101) reported that a 68-year-old female NSCLC patient receiving nivolumab developed autoimmune myocarditis. The patient's electrocardiogram showed sustained ventricular tachycardia and ectopic ventricular beats (101). In addition to the use of corticosteroids for the treatment of myocarditis, other immunosuppressive agents such as anti-thymocyte globulin, infliximab and mycophenolate can also be added if necessary (Figure 2).

## Prevent or Reduce the Frequency of Adverse Events

### Potential Predictive Biomarkers Related to Adverse Effects

The effective management strategy for irAEs is early detection and early intervention. Therefore, it is crucial to find biomarkers that can predict the occurrence of AEs during immunotherapy (102). Recently, a study performed by Kurimoto and his colleague found that serum thyroglobulin, thyroid autoantibodies and early changes in the levels of certain cytokines (increased levels of IL-1β, IL-2, and GM-CSF and decreased levels of IL-8, G-CSF, MCP-1) may indicate the development of autoimmune thyroiditis AEs (103). Similarly, thyroid peroxidase (TPO) and thyroglobulin antibody levels are associated with hypothyroidism in NSCLC patients receiving nivolumab treatment (104). Oyanagi et al. (105) reported that the increase in serum protein RANTES is a potential predictive biomarkers of the onset of irAEs in NSCLC patients who treated with nivolumab. In addition, the increase levels of serum C-reactive protein (CRP) are associated with a higher incidence of irAEs, but not with the severity of irAEs and the affected organ (106). For rare but severe immune-mediated myocarditis, several potential predictive biomarkers have also been found, such as serial troponin, miR-30c (107, 108).

### Baseline Examination Before Immunotherapy Initiation

By comparing the changes of certain biochemical indicators and imaging features of tissues and organs before and after immunotherapy, it can help clinicians to quickly judge any irAEs that may occur (109). Routine baseline assessments include physical examination (height, weight, heart rate, blood pressure, and other general symptoms), imaging examination (chest CT, brain MRI) as well as laboratory tests (blood routine, blood biochemistry, blood glucose, total bilirubin, TSH, free T4, LH, FSH, testosterone, cortisol, ACTH, infectious disease screening, etc.) (109). In addition, carefully ask patient and family the history of autoimmune disease, infectious disease and organ specific diseases are necessary. Clinicians also need to inform patients of potential side effects of immune checkpoint blockade therapy, whether during or after treatment (73). Patients should also promptly feedback any new symptoms of discomfort.

## Personalized Management

Tumor patients of different races, genders, and ages experience different irAEs profiles and severity, therefore precise care according to the patient's personal situation is conducive to reduce the incidence of AEs (110). Elderly people with lung cancer usually have comorbidities and polypharmacy, therefore adequate clinical monitoring is required (110). However, Hakozaki et al. (111) showed that polypharmacy was not associated with irAEs but was associated with higher rate of unexpected hospitalizations during anti-PD-1/PD-L1 treatment in early NSCLC patients (aged ≥ 65 years) in Japanese. Studies have also shown that immune-related fatigue is more common in elderly patients with lung cancer (aged ≥75 years) (49.1 vs. 40.2%), but no other differences in irAEs are observed, and it is not recommended to adjust the dosage of elderly patients (109, 110). Given the small number of elderly patients involved in most immune checkpoint blockade studies, the toxicity data for this group is limited and further studies are needed (112). PD-1/PD-L1 blockade may aggravate or reactivate certain existing viral infectious diseases, therefore patients with a history of chronic viral infections (such as HBV, HCV or HIV) should be excluded from clinical trials (109). Due to the ability of IgG to cross the placental barrier, ICI is not recommended for pregnant and lactating women unless the clinical benefit of the patient outweighs the potential risk (109). Most initial clinical trials of PD-1/PD-L1 blocking therapy are conducted in Caucasians or mix races (113). In recent years, more and more clinical trials of anti-PD-1/PD-L1 agents have been conducted in Asian populations (113). The analysis results of Yang et al. (113) showed that in cancer patients with PD-1/PD-L1 blockade therapy, the AEs of any grade with different prevalences between Asian populations and Western/international populations included fatigue, diarrhea, nausea, rash, vomiting, and hypothyroidism. Overall, we still need to develop more sophisticated medical tools in the future to achieve the best management strategy for irAEs in cancer patients.

## Conclusion

The therapy based on PD-1/PD-L1 immune checkpoint blockade show a better tolerated than traditional standard chemotherapy in NSCLC patients, but the AEs of these drugs are different from traditional cytotoxic therapy. Therefore, it is necessary to increase awareness of these treatment-related toxic reactions for better management. These adverse reactions involved different tissues and organs in the human body, causing toxic reactions ranging from mild fatigue to severe, life-threatening liver and lung toxicity (115, 116). Compared with traditional chemotherapy, AEs caused by anti-PD-1/PD-L1 treatment were usually of low grade, with relatively good patient tolerance and fewer deaths. However, due to the rapid onset of AEs, so timely medical care was crucial, especially for the elderly patients, these toxic reactions should be more carefully monitored to prevent possible complications.

In conclusion, our review summarizes common and rare adverse reactions based on anti-PD-1/PD-L1 therapy in the treatment of NSCLC. Overall, adverse reactions caused by anti-PD-1/PD-L1 immunotherapy were usually low-grade and most patients were better tolerated. However, there were still some serious and even life-threatening adverse events related to treatment. Therefore, healthcare workers should be alert to the occurrence of such AEs to better monitor and manage these adverse reactions.

## Author Contributions

CS, HW, and YL wrote the first draft of the manuscript. QG, LZ, JL, and WZ organized the structure of the manuscript. JZ, XZ, and YY contributed conception of the work. All authors have read and agreed to the published version of the manuscript.

## Conflict of Interest

The authors declare that the research was conducted in the absence of any commercial or financial relationships that could be construed as a potential conflict of interest.
